# Utility of synovial calprotectin lateral flow test to exclude chronic prosthetic joint infection in periprosthetic fractures: a prospective cohort study

**DOI:** 10.1038/s41598-022-22892-9

**Published:** 2022-11-01

**Authors:** Igor Lazic, Alexander Burdach, Florian Pohlig, Rüdiger von Eisenhart-Rothe, Christian Suren

**Affiliations:** Department of Orthopaedic Surgery, Technische Universität München, School of Medicine, Klinikum rechts der Isar, Klinik für Orthopädie und Sportorthopädie, Ismaninger Str. 22, 81675 Munich, Germany

**Keywords:** Biomarkers, Diagnostic markers, Infectious diseases

## Abstract

The diagnosis of periprosthetic joint infection (PJI) requires a combination of various clinical, laboratory, microbiological and histopathological parameters. A concomitant periprosthetic fracture (PPF) further complicates the diagnosis as it causes a confounding local inflammatory response. Synovial calprotectin has been demonstrated as a promising biomarker of PJI. The purpose of the present study was to evaluate the reliability of synovial calprotectin for the pre- or intraoperative diagnosis of PJI in PFF. 30 patients with PPF and implant loosening were included in this prospective study. Synovial fluid with white blood cells and percentage of polymorphonuclear neutrophils, serum C-reactive protein, and synovial calprotectin using a lateral-flow assay were tested against the EBJIS definition with adjusted thresholds to account for the local inflammation. 14 patients were postoperatively classified as confirmed infections (ten total hip arthroplasties and fourtotal knee arthroplasties). The calprotectin assay yielded a sensitivity of 0.71 [0.48; 0.95], a specificity of 0.69 [0.46; 0.91], a positive predictive value of 0.67 [0.43; 0.91] and a negative predictive value of 0.73 [0.51; 0.96]. Calprotectin is a promising diagnostic parameter for the detection of a PJI in a PPF. The lateral flow assay offers prompt results, which may further assist the surgeon in addition to already existing parameters of PJI diagnostics to diagnose concomitant PJI in PPF during surgery.

## Introduction

Periprosthetic joint infections (PJI) and periprosthetic fractures (PPF) are both devastating complications of total hip arthroplasties (THA) and total knee arthroplasties (TKA)^1–3^. Following aseptic loosening, PJI represents the second most common cause for revision with 14.8% in THA and 25.2% in TKA^4^. Furthermore, it represents the most common cause for total knee revision arthroplasty within the first two years^5^. The cumulative incidence ranges between 0.5 and 3% in THA and TKA^5–8^. On the other hand, PPF has an incidence of 0.1–18% in THA and 0.3 to 5.5% in TKA, respectively^9^. Interestingly, concomitant PJI is present in 12–29% of PPF^3,10,11^. Loosening is known to be a risk factor for PPF, so it is plausible that septic loosening also contributes to PPF^12^. The diagnosis of PJI requires a combination of clinical, laboratory, pathology, microbiology, and imaging studies^13,14^. In the setting of a PPF, the diagnosis of PJI becomes even more complex: The sensitivity of the currently recommended parameters for PJI like serum C-reactive Protein (CRP) or synovial white blood cell count (WBC) decreases, because a PPF causes a hemarthrosis with a subsequent local inflammatory response, which may confound synovial analyses^15^. However, the appropriate and timely diagnosis of concomitant PJI is of tremendous importance, as the treatment strategies of PJI and PPF are contrary to each other: Chronic PJI requires the exchange of implants with a thorough and radical surgical debridement, whereas in PPF additional fixation devices are implanted and the soft tissue is being preserved as it is already compromised by the trauma itself. Pre- or intraoperative certainty about the presence of a concomitant PJI is therefore crucial. However, postoperatively available results such as microbiological and histopathological findings, which are highly relevant in current definitions of PJI, cannot be adequately considered. Hence, the currently recommended diagnostic algorithms are not equally applicable in PPF and often fail to provide a conclusive preoperative result.

Synovial calprotectin is a proinflammatory protein widely used for the evaluation of chronic inflammatory pathologies and has been demonstrated as a promising biomarker of PJI in TKA, recently^16^. Calprotectin is also known as MRP8/14 or S100A8/A9. It is a heterodimeric complex of two S100 calcium-binding proteins: myeloid-related protein 8 (MRP-8 or S100A8) and myeloid-related protein (MRP-14 or S100A9). It is an important proinflammatory factor of innate immunity acting as an endogenous damage-associated molecular pattern molecule via toll-like receptor 4 activation and is released from activated granulocytes and macrophages during inflammation^16^. Using a lateral flow assay, calprotectin can be analyzed intraoperatively within 15 min, making it suitable for immediate decision making during revision surgery. The purpose of this study is to evaluate the reliability of calprotectin in the diagnosis of concomitant PJI in PPF.

## Methods

### Study design and population

We prospectively included all patients treated for PPF of total hip or knee arthroplasties at our institution between January 2019 and September 2021 classified as Paprosky/Della Valle type 3B, Vancouver type B2 or B3, Lewis-Rorabeck type III or Felix type IB-IVB with intraoperatively confirmed component loosening^17–20^. A total of 30 cases were included. Cases missing both the synovial fluid and the histopathological analysis were not included (n = 2; 7% of cohort). The mean patient age at the time of PPF was 72.3 ± 14.8 years. 21 women and nine men were analyzed. Seven PPF occurred after TKA and 23 PPF occurred after THA. The patient demographics are summarized in Table 1. The study was evaluated and done in accordance with the guidelines approved by the ethics commission of the Technical University of Munich under reference no. 26/19 S-SR. Informed consent was given by all patients. CRP was obtained during the routine preoperative diagnostic workup. All joints were aspirated intraoperatively under aseptic conditions to measure the WBC and differential, including the percentage of polymorphonuclear neutrophils (PMN). As per standard procedure, synovial fluid in blood culture flasks and five tissue biopsies were retrieved for conventional culture and the explanted components were sent for sonication and subsequent culture of the sonication fluid. All cultures were incubated for a minimum of 14 days. One tissue biopsy was sent for pathological classification according to the criteria established by Morawietz and Krenn^21^*.*

**Table 1:** Patient Demographics.

	Septic cases (n = 14)	Aseptic cases (n = 16)
Age, years (mean ± SD)	71.8 ± 14.0	72.7 ± 15.1
Male, n (%)	4 (28.6)	5 (31.3)
THA, n	10	13
TKA, n	4	3
BMI, kg/m^2^ (median (range))	25.4 (16.8–39.1)	24.7 (17.0–39.5)
ASA Score, grade (median (range))	2 (2–3)	3 (2–4)
number of surgeries after primary arthroplasty, n (median (range))	1 (0–3)	1 (0–5)

*THA* Total Hip Arthroplasty; *TKA* Total Knee Arthroplasty; *BMI* Body Mass Index; *ASA*, American Society of Anaesthesiologists.

### Measurement of Calprotectin Assay

The calprotectin lateral flow test (Lyfstone AS, Lysaker, Norway) was subsequently performed according to the manufacturer's specifications. The test results were photometrically evaluated after 15 min using a smartphone application provided by Lyfstone for this purpose. The quantitative read out ranges between 14 and 300 mg/ml calprotectin and the manufacturer proposes three different risk stratification groups to assess the risk of a PJI: low risk (< 14 mg/ml), moderate risk (14–50 mg/ml) and high risk (> 50 mg/ml). The threshold for a PJI was set at 50 mg/ml, as recommended by the manufacturer per se. The surgeons performing the arthroplasty revision were blinded for the calprotectin test results, which were not included in the diagnostic or therapeutic decision process.

### Outcome evaluation

For the outcome evaluation, the cases were classified regardless of the clinical diagnosis as either infected or aseptic based on a modified version of the EBJIS definition of PJI, also considering the postoperative findings^22^. To account for the local inflammation caused by PFF, the threshold for WBC, PMN and CRP were adapted according to van den Kieboom et al.^11^. Hence, a case was classified as a ‘confirmed infection’ if at least one of the following findings was positive: presence of a sinus tract, WBC > 4.55 G/L, PMN > 79%, microbiological growth in two samples with the same pathogen or in sonication fluid with > 50 CFU/ml or presence of type II or III membranes by Morawietz and Krenn^21^ in the histological work up. A case was classified as ‘infection likely’ if two of the following findings were positive: CRP > 1.67 mg/dl, WBC > 1.5 G/L, PMN > 65%, microbiological growth in a single sample or in sonication fluid with > 1 CFU/ml or presence of more than five neutrophils in ten HPF in the histological work up.

### Statistical analysis

Mann–Whitney-U test was used to analyze the statistical significance of data with non-Gaussian distribution. Fisher’s exact test was used to calculate the confidence intervals for sensitivity, specificity, positive predictive value (PPV) and negative predictive value (NPV). The receiver operating characteristic curve (ROC) and area under the curve (AUC) and accuracy were used to compare the diagnostic efficacy. The optimal threshold was determined according to Youden’s Index (sensitivity + specificity − 1). Values of α < 0.05 were considered to indicate statistical significance. All statistical calculations were performed using SPSS Statistics (Version 22.0. IBM Corporation, Armonk, NY).

### Ethics approval and consent to participate

This study was approved by the ethics commission of the Technical University of Munich under no. 26/19 S-SR. Informed consent was obtained from all patients included in the study.

## Results

14 cases were classified as confirmed infections (ten THA and four TKA): Five cases were considered infected due to multiple criteria: two cases with positive histology and microbiological growth of *Staphylococcus saccharolyticus* and *Staphylococcus warneri* in the sonication fluid, respectively. Another case with elevated WBC and microbiological growth of *Staphylococcus epidermidis* in the culture of sonication fluid*,* one case with elevated WBC, PMN and positive histology and another case with elevated WBC and PMN. Six cases were considered infected due to positive histology alone. One case was considered infected due to elevated WBC. Another case was considered infected due to elevated PMN and a further one was considered infected due to a sinus tract.

Five cases were classified as likely infected (four THA and one TKA): One case was likely infected due to elevated WBC and a single positive culture with *Staphylococcus aureus*. Another case was ruled septic due to elevated PMN and CRP levels. Three further cases had microbiological growth and elevated CRP levels: one case showed growth of *Micrococcus luteus* in the culture of the sonication fluid, another case showed a single positive culture with *Staphylococcus capitis,* and the third revealed two single positive cultures with two different pathogens (*Eggerthella lenta* and *Staphyloccus haemolyticus)*. The synovial fluid analysis was not feasible in 14 out of 30 cases due to clot formation in the sample because of extensive blood contamination in the aspirate.

### Diagnostic performance of preoperatively available EBJIS 2021 definition criteria and the calprotectin assay

**Figure 1:**
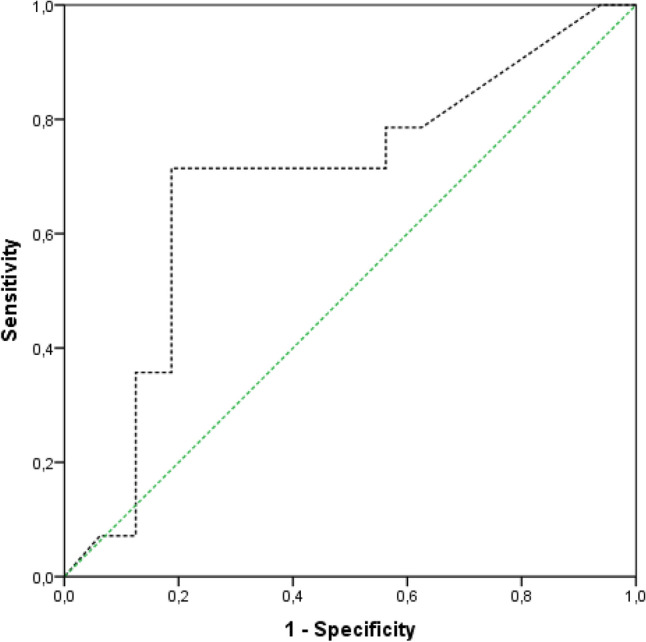
Area under the receiver operating characteristic (ROC) curve (AUC) of the calprotectin lateral flow assay against the modified EBJIS 2021 definition. AUC = 0.69; EBJIS 2021, European Bone and Joint Infection society definition of periprosthetic joint infection published in 2021.

The performances of the preoperatively available criteria of the calprotectin lateral flow test and of a combination of both tests were statistically measured with the modified EBJIS 2021 definition serving as the gold standard. The results are demonstrated in Table 2. 6 out of 14 confirmed infections were evident using the preoperatively available EBJIS 2021 definition criteria. These included the four cases considered infected due to elevated WBC, one case with a sinus tract and another case with elevated PMN. One of two cases preoperatively considered likely infected due to elevated PMN and CRP levels turned to a confirmed infection due to positive histology and growth of *Staphylococcus warneri* in the sonication fluid. 10 out of 14 confirmed infections were identified using the calprotectin lateral flow assay. Calprotectin levels were true positive in three of five cases considered infected due to multiple findings. Furthermore, calprotectin levels were true positive in four of six cases considered infected due to indicative histology alone. The cases considered confirmed infection solely due to WBC, PMN and a sinus tract were all true positive for calprotectin. The results are summarized in Table 3. The AUC for the calprotectin test was 0.69, with an optimal threshold at 76 mg/dl calprotectin (Fig. 1).

**Table 2:** Performance of preoperative criteria WBC, PMN, CRP, clinical features, calprotectin lateral flow assay, microbiological cultures and histopathological results.

	Serum-CRP	WBC	PMN	Preoperative criteria EBJIS 2021	Calprotectin	Preoperative criteria EBJIS 2021 + Calprotectin	Positive microbiology	Positive histopathology
Sensitivity [95% CI]	0.57 [0.29, 0.82]	0.40 [0.12, 0.74]	0.30 [0.07, 0.67]	0.42 [0.14, 0.70]	0.67 [0.40, 0.93]	0.79 [0.57, 1.00]	0.21 [0.04, 0.51]	0.64 [0.35, 0.87]
Specificity [95% CI]	0.50 [0.25, 0.75]	1.00 [0.79, 1.00]	1.00 [0.79, 1.00]	1.00 [1.00, 1.00]	0.79 [0.57, 1.00]	0.75 [0.51, 1.00]	1.00 [0.79, 1.00]	1.00 [0.79, 1.00]
PPV [95% CI]	0.50 [0.25, 0.75]	1.00 [0.40, 1.00]	1.00 [0.29, 1.00]	1.00 [1.00, 1.00]	0.73 [0.46, 0.99]	0.79 [0.57, 1.00]	1.00 [0.29, 1.00]	1.00 [0.66, 1.00]
NPV [95% CI]	0.78 [0.52, 0.94]	0.73 [0.50, 0.89]	0.70 [0.47, 0.87]	0.67 [0.47, 0.87]	0.73 [0.51, 0.96]	0.75 [0.51, 1.00]	0.59 [0.39, 0.78]	0.76 [0.53, 0.92]
Accuracy	0.53	0.77	0.73	0.73	0.73	0.77	0.63	0.83

The EBJIS 2021 definition served as the gold standard; CRP, serum C-reactive Protein, WBC, synovial white blood cell count; PMN, percentage of polymorphonuclear neutrophils in synovial white blood cell count; CI, confidence intervall; PPV, positive predictive value; NPV, negative predictive value; EBJIS 2021, European Bone and Joint Infection society definition of periprosthetic joint infection published 2021.

**Table 3:** Results of calprotectin, serum CRP, synovial WBC, synovial PMN, histology, microbiology and clinical findings of the confirmed infections according to the modified EBJIS 2021 definition of PJI.

Patient	THA or TKA	Calprotectin (mg/ml)	CRP (mg/dl)	WBC (G/L)	PMN (%)	Histology	Microbiology	Sinus Tract
1	THA	14	0.4	1.02	12	Positive	Negative	Negative
2	THA	14	0.2	6	64	Negative	*Staphylococcus epidermidis*	Negative
3	THA	275	5	14	83	Positive	Negative	Negative
4	THA	14	0.2	0.7	58	Positive	*Staphylococcus saccharolyticus*	Negative
5	THA	105	14.6	N/A	N/A	Positive	Negative	Negative
6	THA	28	8.8	N/A	N/A	Positive	Negative	Negative
7	THA	85	1.5	1.01	79	Positive	*Staphylococcus warneri*	Negative
8	THA	119	7.3	N/A	N/A	Positive	Negative	Negative
9	THA	300	21	7.5	64	Negative	Negative	Negative
10	THA	124	0.4	N/A	N/A	Positive	Negative	Negative
11	TKA	217	4.3	1.21	81	Negative	Negative	Negative
12	TKA	96	0.5	0.7	60	Negative	Negative	Positive
13	TKA	172	5.4	0.32	60	Positive	Negative	Negative
14	TKA	256	5.3	8.65	88	Negative	Negative	Negative

THA = Total Hip Arthroplasty; TKA = Total Knee Arthroplasty; N/A = not available; EBJIS 2021, European Bone and Joint Infection society definition of periprosthetic joint infection published in 2021.

### False positive and false negative cases

The calprotectin lateral flow assay showed five false positive and four false negative results. Of the five false positive cases, two had no representative synovial fluid analysis, one case showed a PMN of 76%, and one yielded a calprotectin level of 56 mg/dl, which lies just above the threshold of 50 mg/dl. Two of the four false positive cases were considered likely infected: one case with elevated PMN and CRP levels as well as one case with elevated CRP levels and growth of *Micrococcus luteus* in the sonication fluid. One false positive case showed only elevated CRP preoperatively. Of the four false negative cases, two cases had no positive findings apart from an indicative histology. Another false negative case showed an elevated WBC of 6 G/L with 64% PMN. The last false negative case showed an indicative histology and growth of *Staphylococcus saccharolyticus* in the sonication fluid.

### Synopsis of preoperatively available EBJIS 2021 definition criteria and the calprotectin assay

Using a synopsis of CRP, WBC, PMN, presence of sinus tract and the calprotectin results, 11 out of 14 confirmed infections were identified. The false positive classifications remained as described above. One false negative case (WBC of 6 G/L), however, turned true positive due to the additional information of the additional inflammation biomarkers. Increasing the calprotectin threshold to 76 mg/dl on the basis of the results of this study, two false positive cases would turn into true negative cases (one case with elevated CRP, the other case with no signs of inflammation). This threshold adjustment would therefore yield a sensitivity of 0.71 [CI 0.48; 0.95], a specificity of 0.81 [CI 0.54; 0.96], a positive predictive value (PPV) of 0.77 [CI 0.46; 0.95], a negative predictive value (NPV) of 0.76 [CI 0.50; 0.93] and an accuracy of 0.77, respectively. Applying this threshold adjustment into the synopsis of the biomarkers would result in a sensitivity of 0.79 [CI 0.49; 0.95], a specificity of 0.81 [CI 0.54; 0.96], a PPV of 0.79 [CI 0.49; 0.95], a NPV of 0.81 [CI 0.54; 0.96], and an accuracy of 0.8.

## Discussion

The most important finding in this study is that synovial calprotectin is a promising biomarker to timely differentiate between PJI and aseptic PPF. Calprotectin has been recently introduced as an auspicious synovial biomarker in PJI diagnostics with a highly accurate diagnostic performance. Salari et al. evaluated 76 patients with painful TKA in a prospective study and demonstrated a sensitivity of 1.0 and a specificity of 0.95 for calprotectin using an enzyme-linked immunosorbent assay (ELISA)^16^. The diagnosis of a concomitant PJI in a PPF is, however, more challenging. The early distinction between septic and aseptic cases in this context is of major importance, as it may prevent additional surgical procedures due to overlooked PJI and unnecessary two-staged revisions in actual aseptic cases.

In the current PJI definitions, much importance is assigned to postoperative criteria such as microbiological and histopathological results of intraoperative tissue samples, so that a final classification as septic or aseptic can often only be made *postoperatively*. We would therefore like to emphasize the value of the calprotectin point-of-care test as a strong *pre-* or *intraoperative* biomarker, which may impact decision making during revision surgery. Point-of-care diagnostic tools for calprotectin have already been evaluated in the setting of a PJI^23,24^. Wouthuyzen-Bakker et al. analysed 52 patients with painful knee, hip and shoulder arthroplasties using a lateral-flow assay for calprotectin (originally designed for inflammatory bowel disease) and reported a sensitivity of 0.87 and a specificity of 0.92. Furthermore, Trotter et al. investigated the same PJI-specific calprotectin lateral flow test as in this study. The authors retrospectively evaluated 52 THA and 17 TKA and demonstrated a sensitivity of 0.75 and a specificity of 0.76^24^. In this study, synovial calprotectin yielded comparable results with a marginally lower diagnostic performance using the lateral-flow assay with the standard threshold of 50 mg/dl in the setting of PPF. To our knowledge, this is the first study to investigate the role of calprotectin for diagnosing concomitant PJI in PPF. Furthermore, only a limited number of studies evaluated other inflammatory biomarkers for PJI diagnostics in PPF, at all. However, there is consensus that the diagnostic utility of blood and synovial inflammatory biomarkers for PJI diagnostics is lower in the setting of PPF: elevated inflammatory biomarkers are not reliable predictors of PJI if fractures are involved^3,10,11^. Van den Kieboom et al. retrospectively compared 103 patients with PPF with 41 patients having concomitant PJI. The authors reported a sensitivity of 0.94 and a specificity of 0.4 for CRP, 0.87 and 0.78 for WBC, 0.79 and 0.63 for PMN, respectively. The synopsis of these biomarkers resulted in a sensitivity of 0.84 and a specificity 0.79. In addition, Chevillotte et al. studied 204 PPF, which included 21 cases of concomitant PJI, and concluded that all biomarkers yielded a poor diagnostic performance, even when they were combined^3^. The authors demonstrated a sensitivity of 0.83 and a specificity of 0.56 for CRP, 0.24 and 0.85 for WBC, 0.5 and 0.69 for ESR as well as 0.0 and 0.95 for the combination of all three biomarkers. Furthermore, Shah et al. investigated 121 patients treated for PFF with 14 concomitant PJI and yielded a sensitivity of 0.71 and a specificity of 0.44 for CRP, 0.83 and 0.65 for WBC, 1.0 and 0.63 for PMN as well as 0.86 and 0.40 for ESR, respectively. Hence, these inflammatory biomarkers for PJI show a poor diagnostic performance in the setting of PPF with a high rate of false positives^3,10,11^. On the contrary, the recent diagnostic algorithms for PJI apply low thresholds to further increase sensitivity for low-grade infections. Consequently, the current EBJIS definition of PJI explicitly indicates that the recommended biomarkers used for serum, histopathologic and synovial fluid analysis are not validated in the setting of PPF^22^. To account for this local inflammation, we modified the current EBJIS definition and applied the thresholds for WBC, PMN and CRP as described by van den Kieboom et al.^11^.

In this study, we were able to show that the sensitivity of calprotectin is higher compared to serum-CRP as well as synovial WBC and PMN, whereas WBC and PMN demonstrated great specificity. In this context, however, it must be mentioned that the sensitivity of WBC and PMN may potentially remained low since the conventional synovial analysis was not feasible in ten cases. In view of the literature for WBC and PMN in this regard, it must be assumed that most likely the specificity is lower while the sensitivity is higher for both biomarkers^3,10,11^. Hence, it cannot be finally ascertained whether the diagnostic performance of calprotectin is superior based on this study. However, in clinical reality, obtaining sufficient synovial fluid is often difficult, so that a considerable merit of the calprotectin LFT is that it only requires the smallest amounts of synovial fluid (20 µl). Thus, based on the results of this study, the calprotectin LTF can be considered as an additional promising tool in this particular clinical setting. Interestingly, the synopsis of calprotectin, CRP, WBC, PMN and the clinical signs of infection increased the diagnostic performance considerably in this study, which underlines the potential added value of calprotectin to existing PJI diagnostics in the setting of PFF. In regard of the high specificity of the synovial analysis and the comparably high sensitivity of calprotectin, these tests may serve as rule in and rule out tests, respectively. Similarly, the adjustment of the calprotectin threshold to 76 mg/ml on the basis of our ROC analysis further increased the predictive performance of this novel biomarker, especially resulting in an increased specificity. Based on this threshold adjustment, the diagnostic utility of calprotectin alone is comparable to the diagnostic utility of the combined inflammatory biomarkers as reported by van den Kieboom et al.^11^. Trotter et al. already discussed a readjustment of the calprotectin threshold to improve the test performance in detecting low-grade infections^24^. Similarly, such a readjustment in the other direction might be helpful to adjust for the inflammation caused by the fracture (or other pathologies). Unfortunately, there is no final consensus on the definitive thresholds for calprotectin currently, especially not in the setting of PFF^11,25^. We therefore highlight the low specificity of calprotectin with a threshold of 50 mg/dl and advise to take particular caution as false positive results can likewise cause extensive overtreatment.Eventually, the diagnosis of a PJI in PPF continues to be the subject of research and the validation of calprotectin as a novel biomarker must be critically reviewed under these circumstances.

This study has several limitations. First, concerns may rise to the small size of the cohort. However, to our knowledge, this is the first prospective study to evaluate the calprotectin lateral flow test in the rare setting of a concomitant PJI in PPF. To increase the pretest probability, the cohort was limited to PPF with clinical or radiological signs of component loosening that may indicate pre-existing low-grade infections. More data is required to confirm these results as the confidence intervals are too wide to draw conclusions with high confidence. Second, not all tests were available in every case, which implies the potential risk of overfitting. In 14 out of 30 cases, the synovial fluid analysis failed due to bloody and clotted aspirates. While highly unlikely, this could either lead to an increase of false negative or true positive cases, which then could potentially compromise the reproducibility of the results. Synovial fluid analysis is indeed a relevant cornerstone of PJI diagnostics, but the diagnosis may also be confirmed in its absence. Since the EBJIS definition is a summative definition without obligatory criteria further true positive cases may occur with every further test applied. This gold-standard problem poses a severe dilemma, which, however, is not limited to our study. We therefore refrained to exclude these cases, especially as missing synovial analysis does represent the clinical reality in the setting of suspected PJI in PPF and limited the selection of tests to our usual clinical approach. In this context, a considerable merit of the calprotectin assay is that it only requires the smallest amounts of synovial fluid. With haemarthrosis and subsequent intraarticular clotting present, the aspiration of sufficient synovial fluid required for standard synovial analysis may be complicated, while the acquisition of sufficient fluid for the calprotectin test is certain, if not least intraoperatively. Third, the prevalence of PJI in PPF is reported to lie between 12 and 29%^3,10,11^. In this study, the prevalence was 47%. However, as described, the diagnostic criteria of a PJI in the case of a PPF are not well established and the criteria applied vary in the existing literature. In addition, a PJI can easily be overlooked due to the already elevated inflammatory biomarkers, so that we assume a higher incidence in clinical practice than reported in the literature. Ultimately, it has to be noted that the classification by Morawietz and Krenn differs from the requirements of the EBJIS definition of PJI (23 neutrophils/ ten HPF vs. five neutrophils/ five HPF). However, in this analysis, this discrepancy did not alter the histological results. Likewise, the calprotectin lateral flow test has not been validated in the setting of PPF before.

In conclusion, these results suggest that the calprotectin lateral flow assay is a promising diagnostic test for the detection of a PJI in a PPF. Its diagnostic utility is comparable to a synopsis of the established preoperative biomarkers so that it can be effectively applied as an additional test for PJI diagnostics. In addition, the calprotectin lateral flow assay provides prompt results available for pre-or intraoperative decision making with only a minimal amount of synovial fluid being required. However, the gold-standard problem and the potential increase of true positives must be considered regarding the amount of failed synovial analyses in this cohort. Larger prospective studies are required to define the diagnostic performance of the calprotectin point-of-care test more precisely.

## Supplementary Information

Supplementary Information.

## Data Availability

Data are available from the authors upon reasonable request and with explicit permission of the participants.
