# Supplementary material for: Utility of synovial calprotectin lateral flow test to exclude chronic prosthetic joint infection in periprosthetic fractures: a prospective cohort study
Source: Sci Rep. 2022 Nov 1;12:18385. doi: 10.1038/s41598-022-22892-9 (PMC9626574; doi:10.1038/s41598-022-22892-9)
Supplement: Supplementary file 1 — Supplementary Information. [file 41598_2022_22892_MOESM1_ESM.docx]

| ***Patient*** | ***THA or TKA*** | ***Calprotectin  (mg/ml)*** | ***CRP  (mg/dl)*** | ***WBC  (G/L)*** | ***PMN  (%)*** | ***Histology*** | ***Microbiology*** | ***Sinus Tract*** |
| --- | --- | --- | --- | --- | --- | --- | --- | --- |
|  |  |  |  |  |  |  |  |  |
| 15 | TKA | 14 | 0.1 | 0.79 | 28 | Negative | Negative | Negative |
| 16 | THA | 283 | 12.4 | N/A | N/A | Negative | Negative | Negative |
| 17 | THA | 14 | 6.4 | N/A | N/A | Negative | Negative | Negative |
| 18 | TKA | 300 | 6.1 | N/A | N/A | Negative | Negative | Negative |
| 19 | THA | 67 | 1.8 | N/A | N/A | Negative | Negative | Negative |
| 20 | THA | 41 | 6.5 | N/A | N/A | Negative | Negative | Negative |
| 21 | THA | 42 | 0.3 | 1.7 | N/A | Negative | Negative | Negative |
| 22 | THA | 45 | 0.2 | N/A | N/A | Negative | Negative | Negative |
| 23 | THA | 14 | 0.1 | N/A | N/A | Negative | Negative | Negative |
| 24 | THA | 14 | 9.8 | 0.22 | 36 | Negative | Negative | Negative |
| 25 | TKA | 31 | 4.2 | N/A | N/A | Negative | Negative | Negative |
| 26 | THA | 136 | 3.3 | 0.4 | 76 | Negative | Negative | Negative |
| 27 | THA | 56 | 0.3 | 0.6 | 51 | Negative | Negative | Negative |
| 28 | THA | 13 | 0.3 | 0.32 | 23 | Negative | Negative | Negative |
| 29 | THA | 20 | 0.6 | N/A | N/A | Negative | Negative | Negative |
| 30 | THA | 14 | 0.3 | N/A | N/A | Negative | Negative | Negative |

**Supplement 1: Results of calprotectin, CRP, WBC, PMN, histology, microbiology and clinical findings of the aseptic cases according to the modified EBJIS 2021 definition of PJI.** THA = Total Hip Arthroplasty; TKA = Total Knee Arthroplasty; N/A = not available; EBJIS 2021, European Bone and Joint Infection society definition of periprosthetic joint infection published in 2021.
